# Comparison of pre-filter and post-filter ionised calcium monitoring in continuous veno-venous hemodiafiltration (CVVHD-F) with citrate anti-coagulation

**DOI:** 10.1371/journal.pone.0189745

**Published:** 2017-12-22

**Authors:** Matthew J. Brain, Owen S. Roodenburg, John McNeil

**Affiliations:** 1 Australian and New Zealand Intensive Care Research Centre (ANZIC-RC), School of Public Health and Preventive Medicine, Monash University, Melbourne, Australia; 2 Intensive Care Unit, Launceston General Hospital, Tasmania, Australia; 3 Intensive Care Unit, The Alfred Intensive Care Unit, Melbourne, Australia; Universita degli Studi di Perugia, ITALY

## Abstract

**Background:**

It is widespread practice during citrate anticoagulated renal replacement therapy to monitor circuit ionised calcium (iCa^2+^) to evaluate the effectiveness of anticoagulation. Whether the optimal site to sample the blood path is before or after the haemofilter is a common question.

**Methods:**

Using a prospectively collected observational dataset from intensive care patients receiving pre-dilution continuous veno-venous haemodiafiltration (CVVHD-F) with integrated citrate anticoagulation we compared paired samples of pre and post filter iCa^2+^ where the target range was 0.3–0.5 mmol.L^-1^ as well as concurrently collected arterial iCa^2+^. Two nested mixed methods linear models were fitted to the data describing post vs pre filter iCa^2+^, and the relationship of pre, post and arterial samples.

**Setting:**

An 11 bed general intensive care unit.

**Participants:**

450 grouped samples from 152 time periods in seven patients on CRRT with citrate anticoagulation.

**Results:**

The relationship of post to pre-filter iCa^2+^ was not 1:1 with post = 0.082 + 0.751 x pre-filter iCa^2+^ (95% CI intercept: 0.015–0.152, slope 0.558–0.942). Variation was greatest between patients rather than between circuits within the same patient or citrate dose. Compared to arterial iCa^2+^ there was no significant difference between pre and post-filter sampling sites (F-value 0.047, p = 0.827)

**Conclusion:**

These results demonstrate that there is minimal difference between pre and post filter samples for iCa^2+^ monitoring of circuit anticoagulation in citrate patients relative to the arterial iCa^2+^ in CVVHD-F however compared to pre-filter sampling, post filter sampling has a flatter response and greater variation.

## Introduction

Use of citrate for anti-coagulation in continuous renal replacement therapy (CRRT) is increasing in prevalence after demonstrating advantages in prolonging filter life[1,2] and incorporation into KDIGO recommendations[3]. Integration of citrate delivery and calcium replacement algorithms within renal replacement therapy platforms has simplified therapy delivery[4] however system designs are still evolving and monitoring practices vary[5].

**Fig 1:**
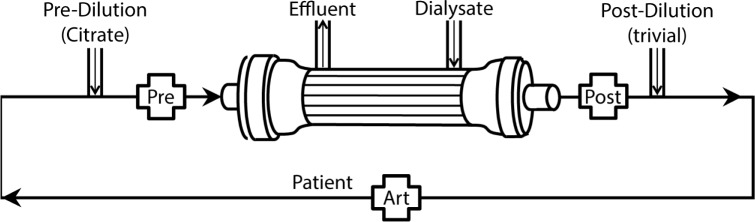
Circuit schematic. Dark arrows denote blood path. Labelled crosses denote sampling ports at pre and post filter and arterial sites.

Detection of effective hypocalcemia by monitoring ionised calcium (iCa^2+^) is accepted to be superior to total calcium measurement[6–10]. A common question from CRRT operators is whether to monitor the effectiveness of citrate by measuring circuit iCa^2+^ from samples taken before or after the haemofilter in the extracorporeal blood path (Fig 1). Manufacturer advice to use post-filter sampling is based on limited evidence[11–13] and it is possible that the most appropriate sampling site may differ between CRRT modality: post filter sampling may be more appropriate for post dilution continuous veno-venous hemofiltration (CVVH) but not necessarily for pre-dilution CVVH or the CVVHD-F modality. Arguably the sampling site should detect the highest [iCa^2+^] values in the extracorporeal circuit so that appropriate increases of citrate dose can occur to augment hypocalcemic anticoagulation at these sites. The samples should also be relatively stable compared to arterial ionised calcium [iCa^2+^] levels to avoid excessive adjustment requirements of citrate dosing.

Calcium ion selective direct potentiometry is the only utilised method for obtaining plasma iCa2+ levels and is a component of many blood gas analysers[14]. Variations measured have been shown to occur at low and high [iCa2+] levels within the analyser and between different analysers [12,15]. In one study these variations were homoscedastic, being more pronounced at low [iCa2+] levels[12], the range that is used to target circuit anticoagulation which raises the possibility that target circuit [iCa2+] may not necessarily indicate sufficient circuit anticoagulation.

The aim of this analysis was to (1) determine if there is any difference between [iCa^2+^] sampled pre-filter or post-filter, (2) establish the extent of variance at pre-filter and post-filter sample sites relative to the arterial [iCa^2+^] and citrate dose, and (3) demonstrate if variance in [iCa^2+^] blood levels between pre-filter and post filter blood samples is the same at low and high concentrations.

## Methods

This was a retrospective post-hoc analysis of a prospectively collected dataset from a prior observational study comparing calcium loss between citrate and heparin[16]. The study was approved by the Tasmanian Network of the Human Research Ethics Committee and included waiving of informed consent due to critical illness with provision of information sheets to patients and relatives. Consecutively admitted adult patients to intensive care who required RRT for at least 12 hours were included. Therapy was implemented by critical care nurses following unit protocols for monitoring and adjustment of citrate. Vascular access was by a 12 French double lumen haemodialysis catheter (Arrow^®^ International) inserted into either the femoral or internal jugular vein. Patient characteristics have been described elsewhere[16].

### CVVHD-F parameters

CVVHD-F was carried out using Baxter^®^ (previously Gambro^®^) Prismaflex™ machines with the Prismaflex ST100™ set containing the AN69ST membrane (Gambro Lundia, Sweden). CVVHD-F for citrate patients was performed with Prismocitrate 10/2™ as pre-dilution and PrismOcal™ as the dialysate and post-blood-pump replacement solution. Patients generally received a blood flow of 200mL.min^-1^ (patient 6 had 15 of 103 observations at 250mL.min^-1^ and patient 8 had only 36 of 120 observations at 200mL.min^-1^ with the remainder being 150-170mL.min^-1^), dialysate flow was generally 2000 ml.hr^-1^ (patient 5 had 15 of 88 observations at 2000mL.hr^-1^, 37 at 1000 mL.hr^-1^ and 36 at 1500mL.hr^-1^ while patient 8 all observations were at 1500mL.hr^-1^). Pre-dilution was initiated at 2000 ml.hr^-1^ (patient 8 was initiated at 1750 mL.hr^-1^). We routinely use 200 ml.hr^-1^ of the dialysate fluid for post filter-replacement to minimise negative pressure in the return line and reduces risk of an air-blood interface in the circuit air-trap.

### Citrate anticoagulation method

Citrate regional anticoagulation was achieved by adjusting the pre-dilution rate of Prismocitrate 10/2 ^TM^ whereby citrate containing pre-dilution fluid is added to the blood path immediately after connection to the vascular access catheter (210 cm prior to pre-filter sampling port on blood path). This was started at 2000 ml.hr^-1^ (equivalent to a citrate dose of 2 mmol citrate.L^-1^_blood_ for a blood flow rate of 200 ml.min^-1^) and adjusted to maintain a pre-filter circuit [iCa^2+^] between 0.3–0.5 mmol.L^-1^. A systemic 10% calcium chloride infusion was infused at 4 ml.hr^-1^ (2.72 mmol.hr^-1^), beginning 15 minutes prior to therapy via a central venous catheter and targeted to maintain serum arterial [iCa^2+^] between 0.8–1.1 mmol.L^-1^. Blood flow, fluid removal rates and filter pressures along with citrate dosage, transmembrane pressures and clinical parameters were recorded hourly.

### Laboratory samples

Samples sets were collected within the first 2 hours of starting CVVHD-F, 2 hourly for the next 12 hours and 4 hourly thereafter until CRRT ceased. Each set consisted of blood taken pre-filter, post-filter and from the patient’s arterial line. One millilitre blood samples were collected into a blood gas syringe (RAPIDLyte™ 3 ml Siemens^®^) and a 0.5 ml blood tube (Capiject^®^ T-M, Terumo Medical Corporation, Elkton, USA). Twenty millilitre effluent samples were collected in a preservative free container. Ionized Ca^2+^ concentration was determined by blood gas analyser (GEM Premier 4000™, Instrument Laboratory) and total calcium analysis of blood and effluent by spectrophotometric dye binding (Abbott Architect c8000, dye for calcium was arsenazo III, analysed at 660 nm).

### Statistical analysis

Statistical analysis was performed in R version 3.2.4[17] with lme4 and nlme mixed effects model packages[18,19] that to account for nested asymmetric repeated measurements. Mixed methods linear models were compared for optimal fit by maximum likelihood estimation with the optimised model then fitted by restricted maximum likelihood estimation. Model goodness of fit was assessed by fitted vs residual plots, quantile-quantile plots for normality and lowest Akaike Information Criterion (AIC) and reported as AIC and likelihood-ratio based coefficient of determination (pseudo-R-squared)[20]. Repeated measures of grouped samples within haemofilter number nested within patients were included as random effects terms. Outlier values were double checked from the original data and retained in model generation, missing values were few and do not affect linear model generation.

Variance breakdown (intraclass correlation) of pre vs post filter iCa^2+^ were extracted from the random effects structure after fitting the mixed model. Models of fit were compared; a standard linear fixed effects model without nesting of repeated measures within circuit within patient (Model 1) and random effects models with a nested structure:

$$y_{k}=\beta_{0}+\beta_{1}x_{k}+\varepsilon_{k}\;k=1,\ldots ,n$$ Model 1

Model 1 was compared to two variants of mixed effects Model 2 and Model 2A:

$$\begin{array}{l} y_{ijk}=(\beta_{0}+b_{0i}+b_{0ij}+b_{2k})+(\beta_{1}+b_{1i}+b_{1ij})x_{ijk}+\varepsilon_{ijk}\\ i=1,\ldots ,7,j=1,\ldots ,7,k=1,\ldots ,n_{ij}\\ b_{i}=\left[\begin{array}{c} b_{0i}\\ b_{1i}\\ b_{2k} \end{array}\right]∼N(0,\sigma_{i}^{2}),b_{ij}=\left[\begin{array}{c} b_{0ij}\\ b_{1ij} \end{array}\right]∼N(0,\sigma_{ij}^{2}),\varepsilon_{ijk}∼N(0,\sigma_{}^{2}) \end{array}$$ Model 2A

Where x_ijk_ and y_ijk_ are respectively the k_th_ pre and post filter iCa^2+^ measurements nested within the j_th_ circuit nested within the i_th_ subject. *β*_*0*_ is the fixed effect intercept coefficient, *β*_*1*_ the fixed effect slope, *b*_*0i*_, *b*_*0ij*_, *b*_*2k*_ describes the variance structure of the intercept random effects (Model 2 lacks the *b*_*2k*_ random effect for citrate dose, where as Model 2A includes this), *b*_*1i*_, *b*_*1ij*_ describes the variance of the slope and ε_ijk_ describes residual variance. A third model (2B, equation not show) included random effects for blood flow, dialysate flow and pre-dilution.

Comparison of pre and post filter results with arterial [iCa^2+^] utilised Model 3 where y_ijkm_ is the [iCa^2+^] value and the fixed effects term *β*_*0m*_ is the intercept for the m^th^ sampling site. *β*_*1m*_ is the slope for [iCa^2+^] at each sampling site through increasing citrate dosage (other terms are as per Model 2):

$$\begin{array}{l} y_{ijkm}=(\beta_{0m}+b_{0i}+b_{0ij}+b_{2k})+(\beta_{1m}+b_{1i}+b_{1ij})x_{ijkm}+\varepsilon_{ijkm}\\ i=1,\ldots ,7,j=1,\ldots ,7,k=1,\ldots ,n_{ij},m=1,2,3\\ b_{i}=\left[\begin{array}{c} b_{0i}\\ b_{1i}\\ b_{2k} \end{array}\right]∼N(0,\sigma_{i}^{2}),b_{ij}=\left[\begin{array}{c} b_{0ij}\\ b_{1ij} \end{array}\right]∼N(0,\sigma_{ij}^{2}),\varepsilon_{ijk}∼N(0,\sigma_{}^{2}) \end{array}$$ Model 3

## Results

### Patient parameters and outcomes

Samples collected from pre-filter, post-filter and arterial sites at 152 time periods (450 samples) from seven patients receiving citrate anticoagulation were included. Patient details and calcium flux have been reported previously[16,21].

### Linear model selection

All the linear models reached significance in describing a fit (F value = 307.7, p<0.001), differences between goodness of model fit by AIC criterion were not statistically significant (Table 1). Of the mixed effects models, slight additional improvement in model fit was achieved by the addition of citrate dose with an adjusted R^2^ of 0.701 (Model 2A). Addition of terms for blood flow, dialysate flow and predilution (Model 2B) lowered AIC at the cost of degrees of freedom without significant improvement in model fit compared to Model 2A consequently Model 2B was utilised for analysis of sources of variance.

**Table 1:** Analysis of variance (ANOVA) table of model fits by maximum likelihood method with significance tests of the mixed effects model fit compared to linear model fit.

	Df	AIC	logLik	deviance	Chisq	F value	p-value	R^2^-LR^2^
Fixed Effect Only Model 1	3	-521.29	263.64	-527.29	NA	307.670	<0.001*	0.680
Mixed Effects Model 2	9	-516.00	267.00	-534.00	6.710	59.232	0.349**	0.694
Mixed Effects Model 2A	10	-517.38	268.69	-537.38	3.382	65.531	0.066**	0.701

Df, degrees of freedom. AIC, Akaike Information Criterion. logLik, log Likelihood. Chisq, Chi-square test. R2-LR2, pseudo R-squared.

* p(>F).

** p(Chisq).

Statistical significance of fit compared to Linear Model 1.

### Model parameters & sources of variance

**Fig 2:**
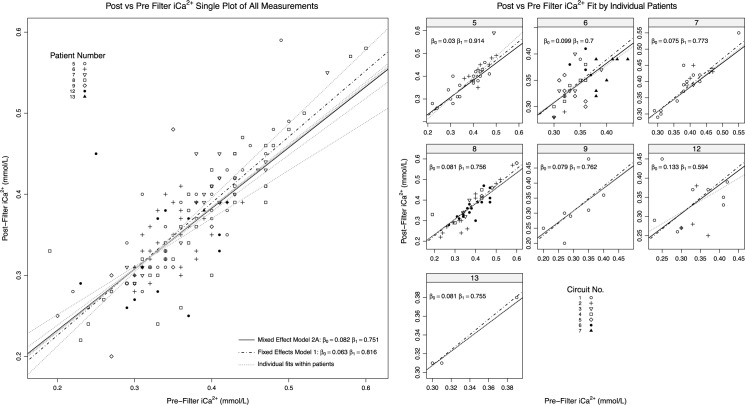
Relationship of post and pre-filter iCa^2+^ samples grouped by: Full dataset (top), and by circuits nested within individual patient/anticoagulation strategy (bottom panels). Trend line is fit from linear mixed Model 2A by each grouping; dotted line in lower panels utilises patient specific intercepts and slopes, a non-mixed effects trend line (Model 1) is also displayed. All outliers are included.

Parameters for model intercepts, slopes and sources of variation for are shown in Table 2 and illustrated in Fig 2. The fixed effect intercept 95% C.I. did not include zero, nor did the slope 95% C.I. include unity. This suggests that pre and post haemofilter [iCa^2+^] observations are unlikely to be interchangeable. Using the means of the fixed effects estimates, pre-filter [iCa^2+^] exceeds post-filter [iCa^2+^] when pre-filter [iCa^2+^] values are above 0.33 mmol/L. Thus pre-filter values generally exceed post-filter values through the target extracorporeal therapeutic range 0.3–0.5mmol/L).

**Table 2:** Fixed effects and sources of variance in nested random effects structure for Model 2A where the independent variable is pre-filter iCa^2+^ and dependent variable is post-filter iCa^2+^. Random effects are assumed to be normally distributed with a mean of zero and S.D. as per values in this table.

Fixed Effect	Effects Estimate	95% C.I.	df	t	p(>|t|)
Intercept	0.082	0.015–0.152	6.242	55.248	<0.001
Pre-Filter [iCa2+]	0.751	0.558–0.942	3.710	8.095	0.002
**Random Effects**	**Model Parameter**	**Intercept / Slope**	**Variance**	**S.D.**	**% Total Variance**
Patient	*b*_*0i*_	(Intercept)	0.0023	0.048	18.5%
	*b*_*1i*_	Pre_Filt_iCa_mmol.L	0.0220	0.148	57.2%
Circuit within Patient	*b*_*0ij*_	(Intercept)	0.0041	0.064	1.1%
	*b*_*1ij*_	Pre_Filt_iCa_mmol.L	0.0299	0.173	10.1%
Citrate.L_BLOOD.Hr	*b*_*2k*_	(Intercept)	0.0002	0.014	0.5%
Residual	*e*_*ijk*_	NA	0.0013	0.036	12.6%

Sources of variance between observations and the fitted model were largely attributable to inter-patient variation (75.7%) with only 11.2% accounted for by different circuits within patients. Variation was larger in model slopes than for intercepts though the sources of this variation were not discernible (Table 2 and Fig 2).

### Comparison of pre-filter and post-filter observations with Arterial iCa^2+^

**Fig 3:**
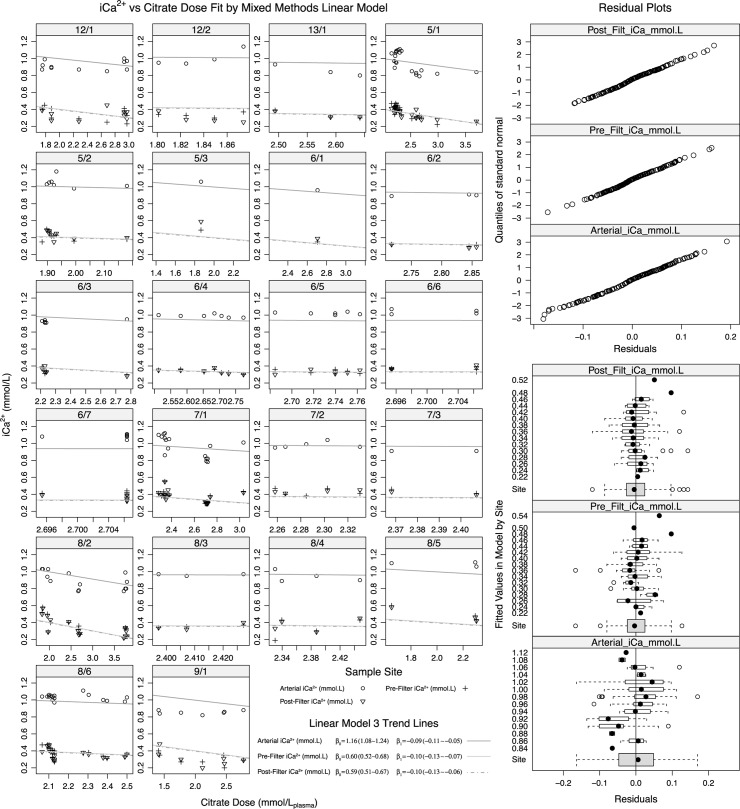
Model 3 fit comparing iCa^2+^ by site and citrate dose by circuits nested within patients. The fit for pre and post filter. β_0_ denotes model intercept, β_1_ denotes slope (iCa^2+^ decrement per unit increase in citrate dose). Quantile residual plots demonstrate variance of fit from normal curve. Fitted residuals (bottom right) graphically illustrate variance across measured observations and by site.

Model 3 was used to compare pre-filter and post-filter values with arterial iCa^2+^ (Fig 3). There was no significant difference between pre and post-filter samples (F-value 0.047, p = 0.827) relative to arterial samples. Inspection of Fig 3 demonstrates that observations from the single circuit used on patient 9 were notable for being consistently lower than model predicted values, on inspection of other available parameters this patient had consistently lower arterial pH (median 7.26, IQR 7.26–7.28) compared to the remaining sampled group (median 7.39, IQR 7.34–7.43). Addition of arterial pH and albumin to Model 3 did not improve fit to the data (p = 0.3564).

Visual inspection of residual plots (Fig 3, lower right) does not suggest significantly different variance across the range of observation levels (pooled pre, post and arterial measurements) however overall variance was significantly (p<0.0001) different between pre, post and arterial sites. Similarly residual variability did not demonstrate a relationship to citrate dose.

## Discussion

We demonstrated minimal difference between choice of monitoring site of [iCa^2+^] for determining extracorporeal circuit anti-coagulation in CVVHD-F with blood and fluid flows typical of adult CRRT. The two sampling sites cannot be considered fully interchangeable as the post-filter site has a flatter response (slope) relative to the pre-filter site resulting in pre-filter values being fractionally higher over much of the target therapeutic range of 0.3–0.5mmol/L. This flatter response may result from the dialysis of calcium and citrate molecules across the haemofilter in different ratios that alters the remaining post filter [iCa2^+^]. The greater variance in response slopes also suggests other patient and hemofilter variables affect post filter [iCa2^+^] to different extents. We thus suggest that in CVVHD-F, sampling pre-filter iCa^2+^ may be more likely to detect concentrations approaching or exceeding the upper therapeutic anticoagulation target within the haemofilter than post-filter samples.

These findings are relevant in that little evidence supports advice to utilise post-filter sampling over pre-filter sampling. It is conceivable that post-filter sampling may be more appropriate in post-dilution CVVH modalities whereas pre-filter sampling may be a more optimal monitoring site in CVVHD-F. This requires further investigation as to which site provides the optimal detection of elevated extra-corporal values and minimises positive feedback of inappropriate citrate dosage adjustment.

Of interest was the higher than expected level of between patient variation in the correlation of pre and post-filter samples. It is not possible to ascertain from this data set if there were different staff / sampling techniques within patients however this seems improbable as multiple shifts care for each patient and equipment and consumables were not altered over the study period. Given our low number of patients the sources of variation cannot be explored but may result from the diverse pathologies enrolled.

We did not observe significant heteroscedasticity between low and normal range iCa^2+^ values which we postulated might exist after the findings of Schwarzer [12] that suggest different analysers may vary in calibration outside the normal physiologic [iCa^2+^] range. Our samples were only exposed only to one analyser and in our case demonstrated wider variation at higher values.

Limitations of this data include the lack of standardised conditions for testing and the lack of a true gold standard test for ionised calcium as described elsewhere[12]. Patient numbers were low which may over-emphasise between patient variation ahead of inter-sample variation. As CVVHD-F was performed with limited variation in blood and fluid flow rates other than alterations of pre-dilution rate (and hence convective clearance) to effect citrate dosage changes we cannot be confident our findings are generalizable to all blood and fluid flow combinations. A much larger dataset with numerous measurements at many different combinations of blood, pre-dilution and dialysate flows would be necessary to evaluate whether these findings are generalizable however the inclusion of citrate dose in the linear models utilised provides a degree of robustness as it is likely to be cross-correlated with variations in pre-dilution and thus convective clearance. Similarly we cannot comment on CRRT performed with significantly higher post-filter dilution rather than pre-filter as used here.

Due to these factors although we suggest that there exists little operational difference between pre and post filter sampling we cannot recommend pre-filter over post-filter sampling without a larger data set or study designed to test one method against another in preventing filter clotting.

In summary for CVVHD-F with pre-dilution using citrate anticoagulation, pre and post-filter ionised calcium monitoring of circuit anticoagulation can be considered equivalent relative to arterial ionised calcium.
